# Effects of Milling Time, Zirconia Addition, and Storage Environment on the Radiopacity Performance of Mechanically Milled Bi_2_O_3_/ZrO_2_ Composite Powders

**DOI:** 10.3390/ma13030563

**Published:** 2020-01-24

**Authors:** May-Show Chen, Hsiu-Na Lin, Yu-Chun Cheng, Alex Fang, Chin-Yi Chen, Pee-Yew Lee, Chung-Kwei Lin

**Affiliations:** 1 Research Center of Digital Oral Science and Technology, College of Oral Medicine, Taipei Medical University, Taipei 110, Taiwan; mayshowc@hotmail.com (M.-S.C.); tiffanylin1214@gmail.com (H.-N.L.); chencyi@fcu.edu.tw (C.-Y.C.); 2 Division of Prosthodontics, Department of Dentistry, Taipei Medical University Hospital, Taipei 110, Taiwan; 3 School of Dentistry, College of Oral Medicine, Taipei Medical University, Taipei 110, Taiwan; 4 Department of Dentistry, Chang Gung Memorial Hospital, Taipei 110, Taiwan; 5 Department of Optoelectronics and Materials Technology, National Taiwan Ocean University, Keelung 202, Taiwan; ksfd2266@gmail.com; 6 Department of Engineering Technology and Industrial Distribution, Texas A&M University, College Station, TX 77843, USA; gpafang@tamu.edu; 7 Department of Materials Science and Engineering, Feng Chia University, Taichung 407, Taiwan; 8 School of Dental Technology, College of Oral Medicine, Taipei Medical University, Taipei 110, Taiwan; 9 Additive Manufacturing Center for Mass Customization Production, National Taipei University of Technology, Taipei 110, Taiwan

**Keywords:** bismuth oxide, zirconia, mechanical milling, radiopacity, mineral trioxide aggregates

## Abstract

Mineral trioxide aggregate (MTA) typically consists of Portland cement (75 wt.%), bismuth oxide (20 wt.%), and gypsum (5 wt.%) and is commonly used as endodontic cement. Bismuth oxide serving as the radiopacifying material reveals the canal filling effect after clinical treatment. In the present study, bismuth/zirconium oxide composite powder was prepared by high energy ball milling of (Bi_2_O_3_)_100−x_ (ZrO_2_)_x_ (x = 5, 10, 15, and 20 wt.%) powder mixture and used as the radiopacifiers within MTA. The crystalline phases of the as-milled powders were examined by the X-ray diffraction technique. The radiopacities of MTA-like cements prepared by using as-milled composite powders (at various milling stages or different amount of zirconia addition) were examined. In addition, the stability of the as-milled powders stored in an ambient environment, an electronic dry box, or a glove box was investigated. The experimental results show that the as-milled powder exhibited the starting powder phases of Bi_2_O_3_ and ZrO_2_ and the newly formed δ-Bi_7.38_Zr_0.62_O_2.31_ phase. The longer the milling time or the larger the amount of the zirconia addition, the higher the percentage of the δ-Bi_7.38_Zr_0.62_O_2.31_ phase in the composite powder. All the MTA-like cements prepared by the as-milled powder exhibited a radiopacity higher than 4 mmAl that is better than the 3 mmAl ISO standard requirement. The 30 min as-milled (Bi_2_O_3_)_95_(ZrO_2_)_5_ composite powder exhibited a radiopacity of 5.82 ± 0.33 mmAl and degraded significantly in the ambient environment. However, storing under an oxygen- and humidity-controlled glove box can prolong a high radiopacity performance. The radiopacity was 5.76 ± 0.08 mmAl after 28 days in a glove box that was statistically the same as the original composite powder.

## 1. Introduction

Mineral trioxide aggregates (MTAs) are widely used as lateral perforation sealing and root canal filling materials in dentistry [1,2]. Generally, MTA are composed of tricalcium silicate-based cement and bismuth oxide as the radiopacifier [3]. After clinical treatment, the X-ray image is taken to reveal the sealing or canal filling effect that is mainly provided by bismuth oxide within MTA. According to ISO 6876/2001 standard, the minimum radiopacity requirement is 3 mmAl for root canal sealing materials [4]. 

Though bismuth oxide is used as the radiopacifier within MTA, various radiopacifiers have been investigated. For instance, gold, silver/tin alloy, bismuth subnitrate, bismuth carbonate, barium sulfate, calcium tungstate, zinc oxide, zirconium oxide, tantalum oxide, lead oxide, lanthanum oxide, etc. have been attempted to replace bismuth oxide as the radiopacifier within MTA. Most of them exhibit a radiopacity higher than 3 mmAl. Among these radiopacifiers, bismuth oxide, zirconium oxide, and tantalum oxide have been used in the commercial products ProRoot^®^, Biodentine^®^, and BioAggregate^®^, respectively. 

In addition to the research and development of the radiopacifier within MTA, the setting time, handling properties, and tooth discoloration are important issues to be improved. Modification of Portland cement or the replacement of the mixing liquid have been attempted to shorten the long setting time of the original MTA. For instance, spray-pyrolyzed MTA have been used and the setting time was significantly shortened [5]. Calcium chloride, calcium lactate gluconate, and resins were used as the accelerator [6,7,8,9]. The effects of manually and mechanically mixing on the performance of MTA-like cements were evaluated [10,11,12]. Tooth discoloration was investigated by replacing bismuth oxide with other radiopacifiers [13,14]. Moreover, during practical clinical treatment, the usage of microscopy [15,16] or ultrasonic placement [12] can improve the performance of MTA. Excellent reviews concerning the above-mentioned issues [17,18,19,20] and the clinical performance of using MTA in conservative dentistry [21] are available. 

In clinical practice, if the MTA powder is not used in one application, some other concerns, including the powder-to-water ratio and the reliability of the powder, will be important. Different powder-to-water ratios have been investigated and reports show that the MTA properties and setting time may be affected [13,22]. The reliability of the powder after opening the MTA package by dentists during clinical practice have not been investigated.

Bismuth oxide not only can be used as a radiopacifier within MTA [23,24,25] but also as an ionic conductor in solid oxide fuel cells [26,27]. Bismuth oxide exhibits four different crystal structures: α-, β-, γ-, and δ-phases. The monoclinic α-Bi_2_O_3_ phase is stable at room temperature, transforms into a face-centered cubic δ-phase at 729 °C, and melts into a liquid phase at 825 °C. When cooling from high temperatures, two possible metastable phases (tetragonal β- and body-centered cubic γ-phase) can be observed [28,29,30]. In the case of solid oxide fuel cell application, high temperature fcc δ-phase Bi_2_O_3_ is one of the best oxide ionic conductors. Whereas for MTA application, limited information concerning the radiopacity of MTA-like cements prepared by various crystalline phases of bismuth oxide have been reported. 

The metastable β-, γ-, and δ-phases Bi_2_O_3_ are not stable at room temperature. Modification by adding higher valence elements or cations with smaller radii are able to preserve the metastable phases at room temperature. Various oxides, including TiO_2_ [31], SnO_2_ [32], ZrO_2_ [33], HfO_2_ [33], Ta_2_O_5_ [30,34,35,36], Nb_2_O_5_ [26,36], MoO_3_ [36], and WO_3_ [31,36], have been investigated to prepare the metastable bismuth oxide phases. Among these oxides, zirconia is widely used in dentistry due to its high compatibility, mechanical strength, good abrasion resistance, and chemical stability. Zirconium oxide has shown to be a potentially superior radiopacifier and can accelerate the hydration reaction [14]. For instance, metastable Bi_7.38_Zr_0.62_O_12.31_ (β-Bi_2_O_3_ phase) powders have been prepared by sol-gel and coprecipitation processes for dental filling and radiopacifying application [37,38]. Whereas Bi_0.78_Hf_0.59_Zr_0.63_O_3.61_ (δ-Bi_2_O_3_) was prepared by mechanochemical synthesis for oxide ionic conductor application [33]. The mechanochemical synthesis occurs during the high energy ball milling process where the input mechanical energy is absorbed by the milled materials and triggers the reaction [39,40,41]. The high energy ball milling process [42,43,44] is an alternative way to prepare metastable materials. In the present study, the effect of the milling time and zirconia addition during high energy ball milling of bismuth oxide and zirconia powder mixtures was evaluated. The radiopacity performance of MTA-like cements prepared by the as-milled powders were investigated. In addition, the reliability of MTA powder stored at various conditions for different durations was investigated.

## 2. Experimental Procedures

In the present study, commercially available powders, α-Bi_2_O_3_ (99.9%) and zirconia (99.98%), were used as the starting materials for high energy ball milling using a SPEX 8000D shaker ball mill (Fisher Scientific, Ottawa, ON, Canada). Powder mixtures with the desired compositions and hardened chromium steel balls (7 mm in diameter, with a ball-to-powder ratio of 5:1) were canned into a SKH 9 high speed steel vial (40 mm in diameter and 50 mm in height). In order to evaluate the effect of milling time, (Bi_2_O_3_)_95_(ZrO_2_)_5_ powder in weight percentage was milled for 3 h that was interrupted every 5 min for the first 30 min and every 30 min to the end of the 3-h milling. An equal length of time was applied after each interruption to cool down the vial. X-ray diffraction (XRD) was used to reveal the structural evolution during the milling process using a Bruker AXS GmbH-D2 PHASER diffractometer (Billerica, MA, USA) with monochromatic Cu Kα radiation and a nickel filter. Selected XRD patterns were analyzed by the Rietveld fitting method using XRD analysis software EVA (Bruker-AXS Diffrac EVA, Bruker, WI) to determine the phase percentages within the material.

Portland cement (75 wt.%), as-milled composite powder (20 wt.%), and gypsum (5 wt.%) were further mixed by a planetary ball mill (PM100, Retsch, Haan, Germany) for 10 min. The powder was then added with deionized water at a powder to water ratio of 3 to 1, manual mixed, loaded into an acrylic mold (with a diameter of 10 mm and a thickness of 1 mm), and set at 37 °C for 24 h to prepare the MTA-like cements. For each as-milled powder, six MTA-like cements samples (N = 6) were prepared and radiographed using a dental X-ray system (VX-65; Vatech Co, Yongin Si Gyeonggi-Do, South Korea). The dental X-ray was operated at a voltage of 62 kV, 10 mA current density, and exposed for 0.64 s at a focus-film distance of 30 cm. An occlusal radiographic film (Kodak CR imaging plate size 2; Eastman-Kodak CO, Rochester, NY, USA) was used to record the radiographic image where each set of MTA-like cement specimens (N = 6) and an aluminum step-wedge with various thicknesses (2–16 mm at an increment of 2 mm) were exposed simultaneously. An imaging processing software (Image J 1.52a, Wayne Rasband, National Institutes of Health, Bethesda, MD, USA) was used to determine the gray values of each step for the aluminum wedge and the specimens by interpolating the gray value of the specimen with those from the aluminum wedge. The corresponding radiopacity of the MTA-like cements can be obtained. Though the same aluminum wedge was used for radiopacity measurement, slight variations may still exist among different sets of samples. Thus, the radiopacities for each set of samples was normalized according to the aluminum wedge taken at the same time. Student’s paired t-test with a significance level of 0.05 and 0.01 was performed using SPSS version 18.0 software (IBM Corporation, NY, USA) for intragroup analysis to compare the radiopacities of MTA-like cements prepared by composite powders after storing at different environments and durations.

## 3. Results and Discussion

### 3.1. Effect of Milling Time and Zirconia Addition

**Figure 1:**
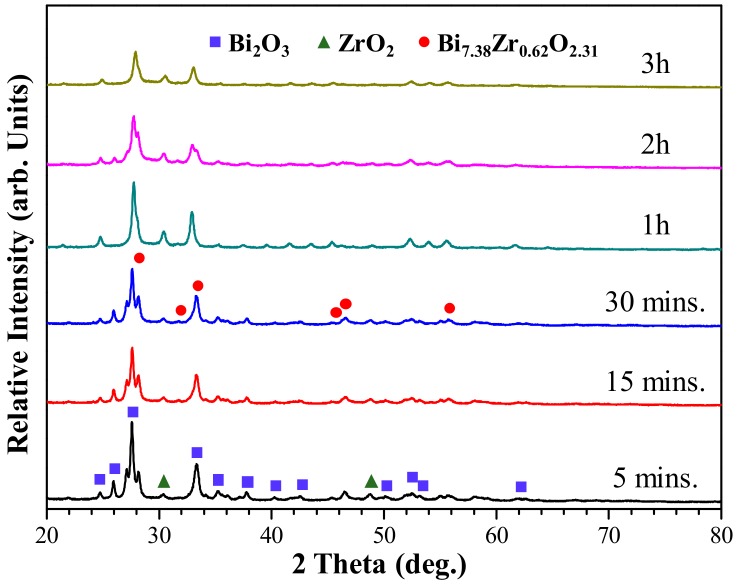
X-ray diffraction patterns of 5% zirconia-added bismuth oxide powder mixture as a.

In the present study, the high energy ball milling process was used to prepare the (Bi_2_O_3_)_100−x_(ZrO_2_)_x_ composite powder that served as the radiopacifier within MTA. Though the radiopacity of a material mainly depends on the atomic number and its density, the variation of the crystalline phase and grain size during the milling process of composite powders may affect the solidification process and the density of MTA-like cements. Figure 1 shows the structural evolution of (Bi_2_O_3_)_95_(ZrO_2_)_5_ composite powder at various milling stages. As revealed by the X-ray diffraction patterns shown in Figure 1, the crystalline peaks of the starting powder Bi_2_O_3_ (ICDD PDF card No. 27-0053) and ZrO_2_ (ICDD PDF card No. 79-1796) continuously decreases with increasing milling time. Whereas, the formation of a metastable δ-Bi_7.38_Zr_0.62_O_2.31_ (ICDD PDF card No. 43-0445) can be observed after merely 5 min of high energy ball milling treatment. The major crystalline phase was Bi_2_O_3_ at the early stage of milling (say up to 30 min) and the metastable δ-Bi_7.38_Zr_0.62_O_2.31_ phase became dominant thereafter (1 to 3 h of milling).

**Figure 2:**
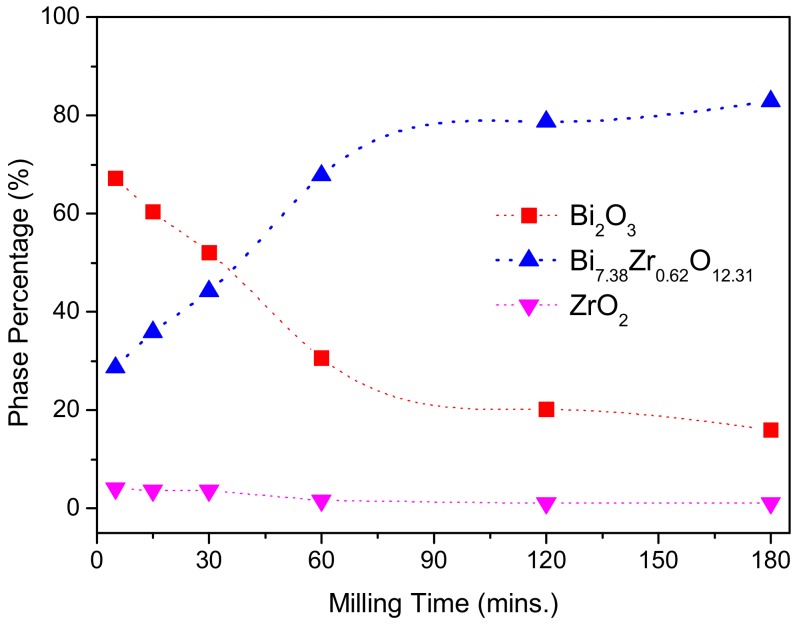
The percentage of the individual phase of (Bi_2_O_3_)_95_(ZrO_2_)_5_ after different milling times.

The phase evolution can be better revealed by the Rietveld fitting analysis [41]. Figure 2 shows the phase percentages of as-milled (Bi_2_O_3_)_95_(ZrO_2_)_5_ composite powder as a function of the milling time. Throughout the high energy ball milling process, Bi_2_O_3_ and ZrO_2_ continuously reacted to form the δ-Bi_7.38_Zr_0.62_O_2.31_ phase. It is interesting to note that the percentage of the metastable δ-Bi_7.38_Zr_0.62_O_2.31_ phase was 28.7% after 5 min of milling. The formation rate roughly exhibited a linear behavior up to 1 h of milling and the amount of the δ-Bi_7.38_Zr_0.62_O_2.31_ phase reached 67.8% for 1 h as-milled powder. Though the percentage of δ-Bi_7.38_Zr_0.62_O_2.31_ phase increased with increasing milling time, the formation rate significantly slowed down after 1 h of milling. At the end of 3 h of milling, the constitutions of the composite powders were δ-Bi_7.38_Zr_0.62_O_2.31_ (82.9%), Bi_2_O_3_ (16.0%), and ZrO_2_ (11.1%). Table 1 summarizes all the crystalline phases of the (Bi_2_O_3_)_95_(ZrO_2_)_5_ composite powder at various milling stages.

**Figure 3:**
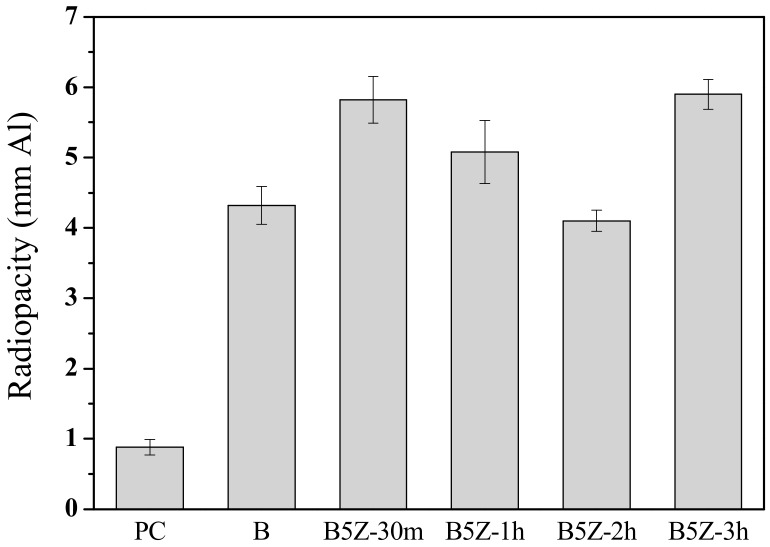
Radiopacities of MTA-like cements prepared by 5% zirconia-added bismuth oxide powder after milling for 30 min, 1 h, 2 h, and 3 h (coded as B5Z-30m, etc.) In addition, Portland cement (sample PC) and MTA with bismuth oxide (sample B) were given as counterparts.

X-ray diffraction analysis revealed that the as-milled (Bi_2_O_3_)_95_(ZrO_2_)_5_ composite powders were a mixture of the starting Bi_2_O_3_ and ZrO_2_ phases and newly formed δ-Bi_7.38_Zr_0.62_O_2.31_ one. In addition, though not examined in the present study, their particle size distribution will be different at various milling stages. Both the phase concentration and the particle size may affect the solidification and the radiopacities of the MTA-like cements. Figure 3 shows the radiopacities of several selected MTA-like cements prepared by as-milled composite powders. The radiopacity was 0.88 ± 0.11 mmAl for Portland cement (sample PC in Figure 3) due to its low atomic number. With the 20 wt.% addition of bismuth oxide (sample B), a significant improvement can be noticed and the radiopacity was 4.32 ± 0.27 mmAl. The B5Z-30m sample (i.e., 30 min as-milled (Bi_2_O_3_)_95_(ZrO_2_)_5_ composite powder) exhibited a radiopacity of 5.82 ± 0.33 mmAl that was among the best radiopacity obtained in the present work. The radiopacity deceased with an increasing milling time up to 2 h. The radiopacity was 5.08 ± 0.45 and 4.10 ± 0.15 mmAl for the B5Z-1h and B5Z-2h samples, respectively. It is suggested that the significant amount of the δ-Bi_7.38_Zr_0.62_O_2.31_ phase may induce difficulty in solidification and is responsible for the decrease in radiopacity. With increasing the milling time to 3 h, the particle size of as-milled powder was fine and the radiopacity reached 5.90 ± 0.21 mmAl, the highest in the present study, but statistically similar to that of the B5Z-30m sample.

**Figure 4:**
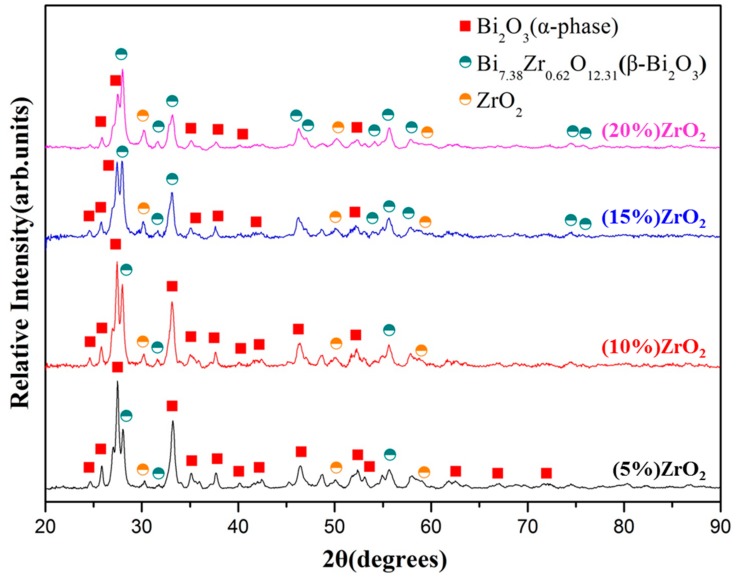
X-ray diffraction patterns of bismuth oxide and zirconia powder mixtures after 30 min of ball milling treatment.

**Figure 5:**
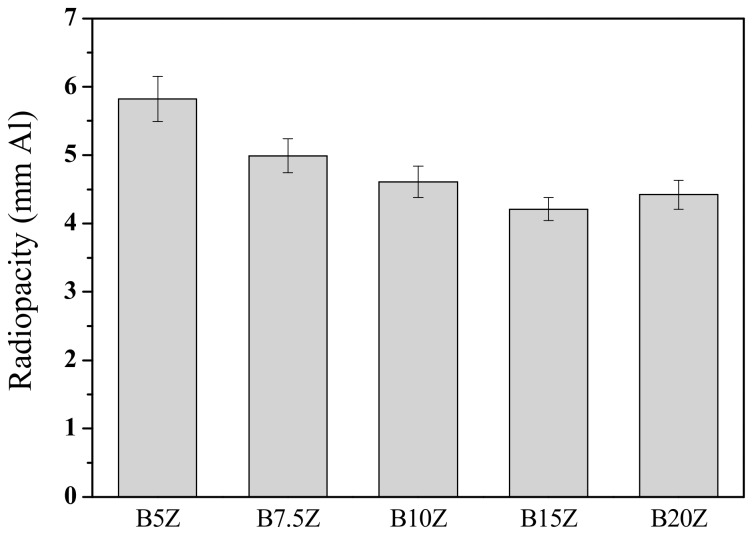
Radiopacity performance of MTA-like cements prepared by various 30 min as-milled zirconia-added bismuth oxide powder into which B5Z, B7.5Z, B10Z, B15Z, and B20Z, indicating 5, 7.5%, 10%, 15%, and 20% zirconia, was added respectively.

The effect of zirconia addition (5–20 wt.%) was investigated by X-ray diffraction and radiopacity investigation. Figure 4 shows the X-ray diffraction patterns of the 30 min as-milled bismuth/zirconia composite powders. It can be noted that the higher the zirconia addition, the larger the amount of the δ-Bi_7.38_Zr_0.62_O_2.31_ phase. The radiopacity, however, decreased with the increasing amount of zirconia addition (up to 15 wt.%), Figure 5. The radiopacity was 5.82 ± 0.33 mmAl for the B5Z sample, monotonically decreased to 4.21 ± 0.17 mmAl for B15Z, and slightly increased to 4.42 ± 0.21 mmAl for B20Z. The decrease in radiopacity may be simply due to the increasing amount of zirconia that has a relatively low radiodensity compared to that of bismuth oxide.

### 3.2. Effect of Storage Environment

**Figure 6:**
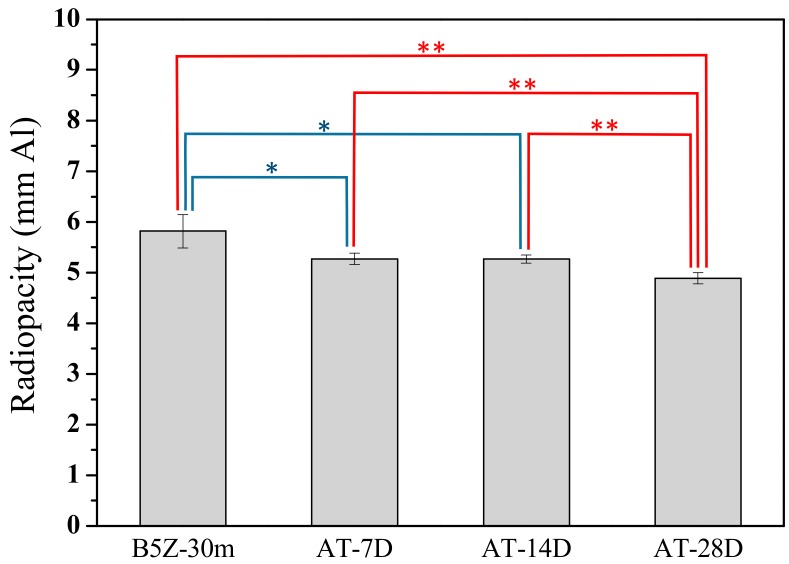
Radiopacity of MTA-like cements stored in an atmospheric environment for 7, 14, and 28 days (i.e., AT-7D, 14D, and 28D, respectively). * and ** indicated that these two sets of samples were statistically different at a 95% and 99% confidence interval, respectively.

During practical application of MTA, sometimes dentists do not use all of the powder after they open the package. In this case, the properties of the MTA powder may be affected by the storage environment. An ambient environment, electronic dry box, and glove box were chosen to simulate the storage conditions. Within the ambient environment where the oxygen and humidity are not controlled, the degradation on the radiopacity performance of the MTA-like cements is quite obvious. As shown in Figure 6, the radiopacity (5.82 mmAl) generally decreased with increasing days within the ambient environment. The radiopacity was 5.27 mmAl after 7 and 14 days in air, but further decreased to 4.89 mmAl at 28 days. The radiopacity was statistically different at a 95% confidence interval even after 7 days. Whereas, the radiopacity after 28 days in the ambient environment was statistically different at a 99% confidence interval when compared with the other samples. 

**Figure 7:**
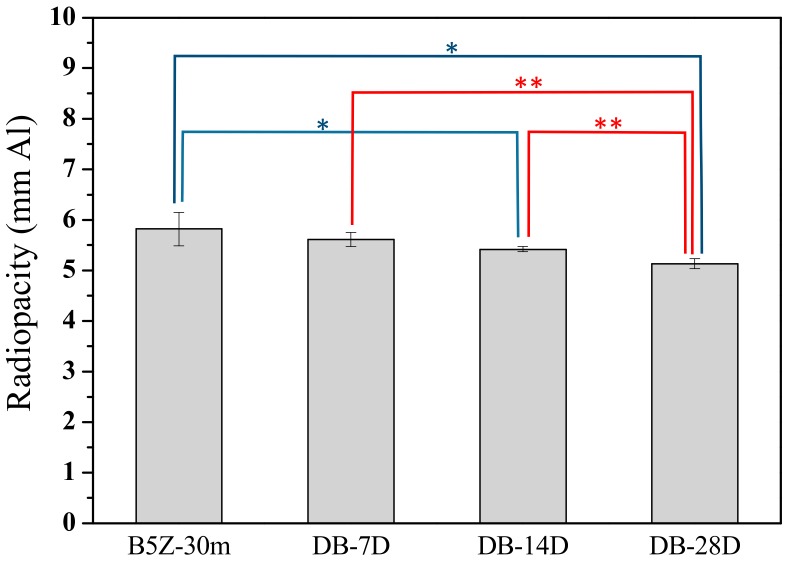
Radiopacity of MTA-like cements stored in an electronic dry box for 7, 14, and 28 days (i.e., DB-7D, 14D, and 28D, respectively). * and ** indicated that these two sets of samples were statistically different at a 95% and 99% confidence interval, respectively.

**Figure 8:**
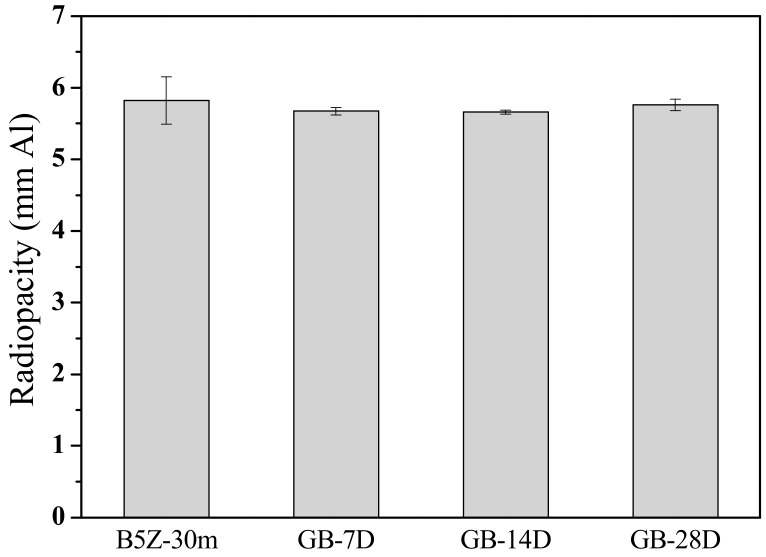
Radiopacity of MTA-like cements stored in a glove box for 7, 14, and 28 days (i.e., GB-7D, 14D, and 28D, respectively).

The degradation on the radiopacity performance was retarded when the MTA was stored within an electronic dry box where the temperature and humidity was controlled. Figure 7 shows the radiopacity of MTA powder stored within a dry box. A continuous decrease in radiopacity with increasing storage time with the dry box can be observed. The radiopacity was 5.82 mmAl and decreased to 5.61, 5.42, and 5.13 mmAl respectively after 7, 14, and 28 days in the dry box. When stored with an oxygen- and humidity-controlled glove box, it is interesting to note that not only did the degradation on the radiopacity almost cease, but the variation (error bars) was also much smaller. As shown in Figure 8, the radiopacity was 5.67 ± 0.05, 5.66 ± 0.03, and 5.76 ± 0.08 mmAl after 7, 14, and 28 days in the glove box, respectively. The radiopacity was statistically similar (no difference, within the standard deviation range) when MTA powder was stored under a well-controlled environment. Table 2 summarizes the radiopacity for MTA-like cements prepared by B5Z-30m (i.e., (Bi_2_O_3_)_95_(ZrO_2_)_5_ as-milled for 30 mins.) powder and stored at various environments after different periods of time.

**Figure 9:**
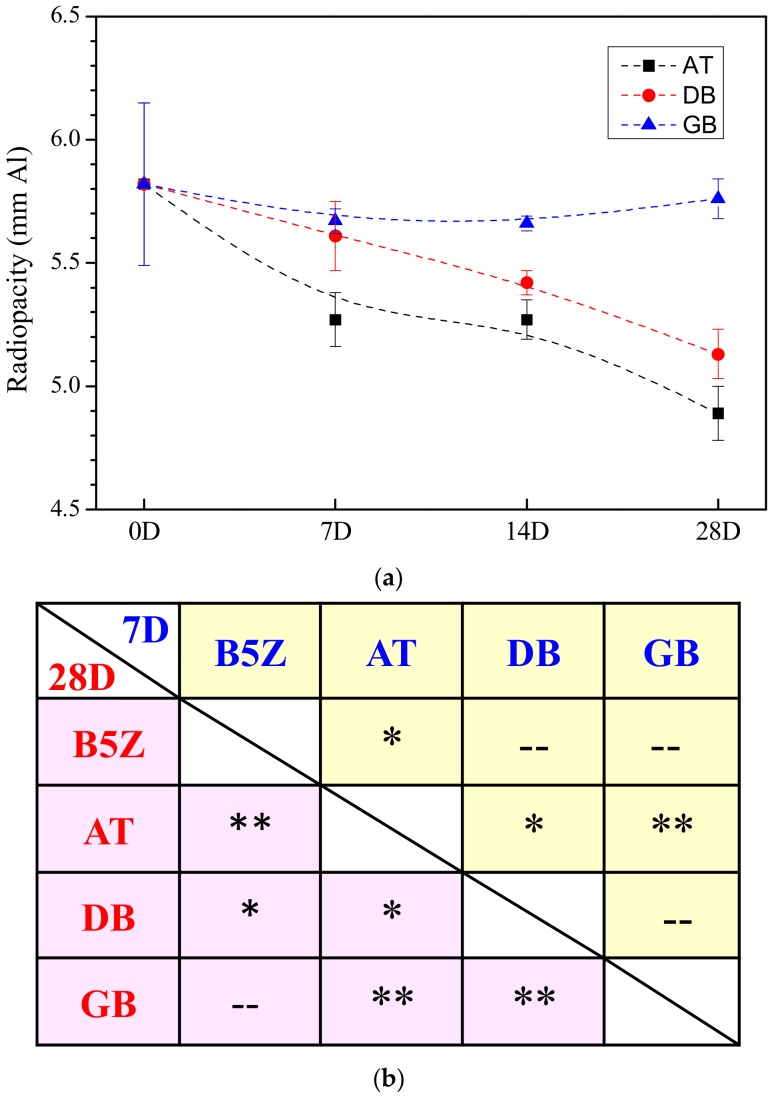
(**a**) Variation of radiopacity and (**b**) comparison between paired samples for MTA-like cements prepared by 30 min as-milled (Bi_2_O_3_)_95_(ZrO_2_)_5_ powder (coded as B5Z) and stored in ambient (AT), dry box (DB), and glove box (GB) environments after 7 days (7D) and 28 days (28D). “--” designated no difference, whereas * and ** indicated that these two sets of samples were statistically different at a 95% and 99% confidence interval, respectively.

In order to better reveal the effect of storage environments, Figure 9 shows the radiopacity for MTA-like cements prepared by the as-milled composite powder and stored at various environments after different periods of time. As shown in Figure 9a, the reliability of radiopacity was the best when stored within a glove box, the MTA powder degraded quickly within the ambient environment, and in between was that retained in an electronic dry box. Figure 9b shows the statistical analysis results where a students’ *t*-test was performed for those stored at various conditions for 7 and 28 days. In the upper right trigonal (comparison among various samples after 7 days), MTA powder stored in air for 7 days (AT) was significantly different (with 95% confidence, marked as *) with B5Z (the counterpart) and that stored within a dry box (DB), and quite different (99% confidence, marked as **) with the glove box one. This indicates that the MTA powder stored in the ambient environment degraded faster than the other two conditions. At the lower left trigonal, the comparison after 28 days of storage, it can be observed that only the one stored within a glove box was statistically similar with the counterpart (B5Z). The rest of the radiopacity results were significantly different at a confidence level of either 95 (*) or 99% (**). This suggests that MTA powder stored at an oxygen- and humidity-controlled environment can prolong the reliability of the radiopacity performance.

## 4. Conclusions

By milling (Bi_2_O_3_)_100−x_(ZrO_2_)_x_ powder mixtures, Bi_2_O_3_ and ZrO_2_ reacted to form the metastable δ-Bi_7.38_Zr_0.62_O_2.31_ phase. The as-milled powder exhibited a mixture of Bi_2_O_3_, ZrO_2_, and δ-Bi_7.38_Zr_0.62_O_2.31_ phases. The longer the milling time or the larger the amount of zirconia addition, the higher the percentage of the δ-Bi_7.38_Zr_0.62_O_2.31_ phase. The MTA-like cements prepared by as-milled composite powder exhibited a radiopacity higher than 4 mmAl (ranged from 4.10–5.90 mmAl) that meets the requirement of ISO standard (3 mmAl). The B5Z-30m (i.e., (Bi_2_O_3_)_95_(ZrO_2_)_5_ as-milled for 30 min) and B5Z-3h (3 h milling) exhibited a similar radiopacity of 5.82 ± 0.33 and 5.90 ± 0.21 mmAl, respectively. In addition, the radiopacity generally decreased with an increasing amount of zirconia addition. The radiopacity was 5.82 mmAl for B5Z and decreased to 4.21 and 4.42 mmAl for B15Z and B20Z, respectively.

The stability of the as-milled powder may vary by the storage environment. When stored under an ambient environment, the as-milled composite powder degraded obviously. The initial radiopacity was 5.82 mmAl, decreased to 5.27 mmAl after 7 and 14 days, and further decreased to 4.89 mmAl at 28 days. The degradation was retarded when the as-milled powder was placed within an electronic dry box and the radiopacity gradually decreased from 5.82 to 5.61, 5.42, and 5.13 mmAl after 7, 14, and 28 days in a dry box, respectively. The reliability of the as-milled powder, however, can be prolonged if it was preserved under an oxygen- and humidity-controlled glove box. After storing it for 28 days in a glove box, the radiopacity was 5.76 ± 0.08 mmAl that was statistically the same as that (5.82 ± 0.33 mmAl) of the original as-milled composite powder. A well-controlled environment can prolong the reliability of the MTA powder.

## Figures and Tables

**Table 1:** Crystalline phases of (Bi_2_O_3_)_95_(ZrO_2_)_5_ as a function of the milling time.

Milling Condition	Milling Time	Crystalline Phases *
Inside Ar-filled glove box	5 min	α-Bi_2_O_3_ (67.2%) + δ-Bi_7.38_Zr_0.62_O_2.31_ (28.7%) + ZrO_2_ (4.1%)
	15 min	α-Bi_2_O_3_ (60.4%) + δ-Bi_7.38_Zr_0.62_O_2.31_ (35.9%) + ZrO_2_ (3.7%)
	30 min	α-Bi_2_O_3_ (52.1%) + δ-Bi_7.38_Zr_0.62_O_2.31_ (44.2%) + ZrO_2_ (3.7%)
	1 h	α-Bi_2_O_3_ (30.7%) + δ-Bi_7.38_Zr_0.62_O_2.31_ (67.7%) + ZrO_2_ (1.6%)
	2 h	α-Bi_2_O_3_ (20.2%) + δ-Bi_7.38_Zr_0.62_O_2.31_ (78.7%) + ZrO_2_ (1.1%)
	3 h	α-Bi_2_O_3_ (16.0%) + δ-Bi_7.38_Zr_0.62_O_2.31_ (82.9%) + ZrO_2_ (1.1%)

*: The percentage of individual phase is given in the bracket.

**Table 2:** Radiopacity for MTA-like cements prepared by 30 min as-milled (Bi_2_O_3_)_95_(ZrO_2_)_5_ powder (coded as B5Z-30m) and stored at ambient (AT), dry box (DB), and glove box (GB) conditions.

Sample Condition	Storage Period ^#^	Radiopacity *
**B5Z-30m**	0D	5.82 ± 0.33
**AT**	7D	5.27 ± 0.11
	14D	5.27 ± 0.08
	28D	4.89 ± 0.10
**DB**	7D	5.61 ± 0.14
	14D	5.42 ± 0.05
	28D	5.13 ± 0.10
**GB**	7D	5.67 ± 0.05
	14D	5.66 ± 0.03
	28D	5.76 ± 0.08

**^#^**: Day is abbreviated as D. 0D-28D indicates different storage period. *: The radiopacity is given as mean ± standard deviation.
